# A seven-gene signature model predicts overall survival in kidney renal clear cell carcinoma

**DOI:** 10.1186/s41065-020-00152-y

**Published:** 2020-09-03

**Authors:** Ling Chen, Zijin Xiang, Xueru Chen, Xiuting Zhu, Xiangdong Peng

**Affiliations:** 1 Department of Gastrointestinal Surgery, Xiangya Hospital, Central South University, Changsha, 410013 Hunan China; 2 Department of Pharmacy, The Third Xiangya Hospital, Central South University, Changsha, 410013 Hunan China

**Keywords:** Kidney renal clear cell carcinoma, Bioinformatics, Prognostic model, LASSO penalty

## Abstract

**Background:**

Kidney renal clear cell carcinoma (KIRC) is a potentially fatal urogenital disease. It is a major cause of renal cell carcinoma and is often associated with late diagnosis and poor treatment outcomes. More evidence is emerging that genetic models can be used to predict the prognosis of KIRC. This study aimed to develop a model for predicting the overall survival of KIRC patients.

**Results:**

We identified 333 differentially expressed genes (DEGs) between KIRC and normal tissues from the Gene Expression Omnibus (GEO) database. We randomly divided 591 cases from The Cancer Genome Atlas (TCGA) into training and internal testing sets. In the training set, we used univariate Cox regression analysis to retrieve the survival-related DEGs and futher used multivariate Cox regression with the LASSO penalty to identify potential prognostic genes. A seven-gene signature was identified that included APOLD1, C9orf66, G6PC, PPP1R1A, CNN1G, TIMP1, and TUBB2B. The seven-gene signature was evaluated in the training set, internal testing set, and external validation using data from the ICGC database. The Kaplan-Meier analysis showed that the high risk group had a significantly shorter overall survival time than the low risk group in the training, testing, and ICGC datasets. ROC analysis showed that the model had a high performance with an AUC of 0.738 in the training set, 0.706 in the internal testing set, and 0.656 in the ICGC external validation set.

**Conclusion:**

Our findings show that a seven-gene signature can serve as an independent biomarker for predicting prognosis in KIRC patients.

## Background

Kidney renal clear cell carcinoma (KIRC) is a type of renal cortical tumour characterized by a growth pattern of the cytoplasm that is associated with malignant epithelial cells and accounts for 80–90% of renal cell carcinomas. In addition, KIRC tends to be resistant to radiation and chemotherapy, which makes surgery the primary treatment [1]. However, 30% of patients who undergo surgery still experience metastasis [2]. Early identification of risk in KIRC patients can help with more accurate clinical treatment. Therefore, there is a strong demand to discover new and reliable markers to predict patient prognosis.

Many studies show that predictive models of gene expression have great significance in clinical prognosis applications. For example, Fatai et al. built a model to demonstrate that a 35-gene signature can discriminate between rapidly and slowly progressing glioblastoma multiforme and predict survival in known subtypes of cancer [3]. Long et al. constructed a prognostic model for patients with hepatocellular carcinoma based on RNA sequencing data [4]. For KIRC, Zhan et al. found that the expression of the five-gene model was related to the prognosis of patients with KIRC by Cox regression analysis [5]. Han et al. analysed reversed-phase protein array (RPPA) data for the protein expression signature of survival time in KIRC [6]. However, the studies of multigene models to predict the prognosis of KIRC patients are still insufficient, and we sought here to use a variety of methods to find more potentially relevant genes.

In terms of survival analysis, Cox proportional hazards regression is currently the most widely used method. However, it is not the most suitable method for high-dimensional microarray data because overfitting is a common shortcoming of modelling using high-dimensional microarray data to identify prognostic genes [7]. The LASSO method can eliminate this limitation and it was applied in our analysis for feature selection [8]. In this study, we sought to identify DEGs associated with OS based on genome-wide expression profiles of KIRC patients [9]. We developed a seven-gene signature by multivariate Cox proportional hazard regression with LASSO penalty [10, 11]. The prognostic model involving these seven DEGs effectively divided KIRC patients into high- and low-risk groups; OS was significantly poorer in the high-risk group than in the low-risk group among the training, testing and ICGC sets. OS was regarded as the endpoint for evaluating the prognostic model and the ultimate measure of treatment benefits [12, 13]. In conclusion, this study may add literature to existing prognostic models of KIRC to identify patients with a higher risk of mortality.

## Results

### Screening for DEGs and GO enrichment analysis

**Fig. 1:**
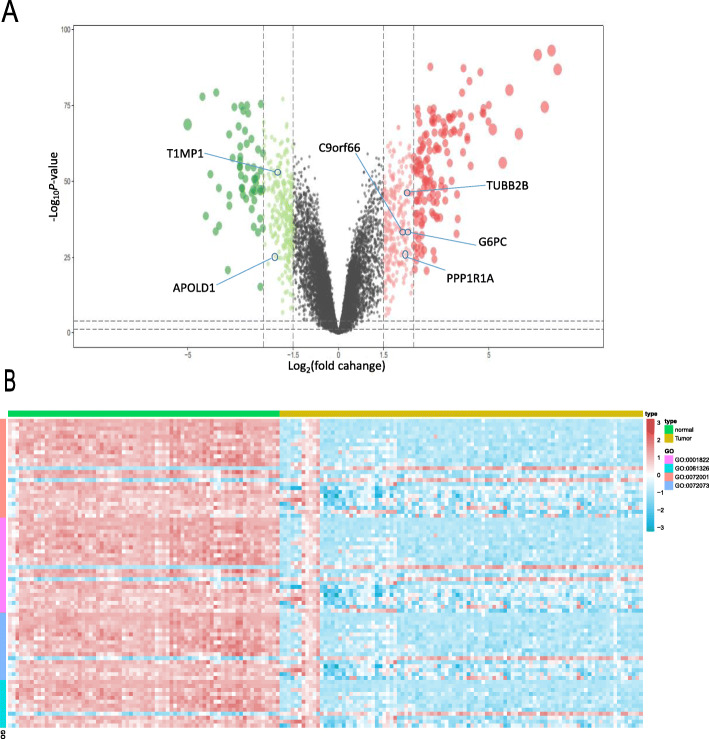
DEGs in KIRC vs adjacent normal tissues. **a**. Volcano plot visualizing the DEGs screened using limma. The red and green points represent the significantly upregulated and downregulated DEGs, respectively (logFC> 2 or logFC<(− 2) with adjusted *P* < 0.05). Features selected by the LASSO penalty are also marked. **b**. Heatmap showing that the 333 DEGs are involved in renal system development, kidney epithelium development, renal tubule development, and kidney development

After GEO data filtering, quality assessment, and data processing, we performed differential expression analysis by using the limma R package and identified 333 DEGs from the GEO cohort.. These DEGs comprised 218 upregulated genes and 115 downregulated genes, using the criteria of logFC > 2 or logFC< (− 2) with adjusted *P* < 0.05 (Fig. 1a). The heatmap in Fig. 1b shows that the 333 DEGs were enriched in 4 nodes. GO analysis revealed that the DEGs were enriched in renal system development, kidney epithelium development, renal tubule development, and kidney development.

### Construction of a prognostic model in the training set

**Fig. 2:**
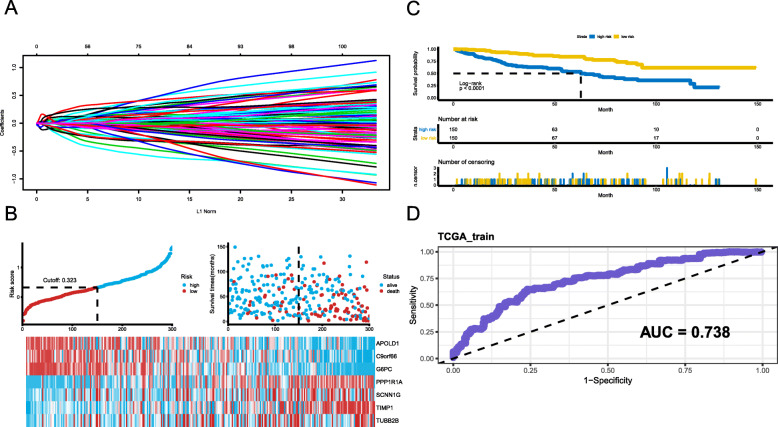
Construction of the KIRC-specific gene risk score system A. LASSO coefficient of the 7 survival-related genes. B-C. Prognostic classifier analysis of the patients in the internal testing set. The distribution of risk score and patients survival time and status, and the lower one is heat map of the genes in prognostic classifier. D. ROC curve for the survival of high- and low-risk groups

DEGs that mediate tumour initiation, progression, and proliferation are potential prognostic biomarkers. To identify potential prognostic DEGs, the TCGA cohort was randomly divided into a training set (*n* = 300) and an internal testing dataset (*n* = 291) with an approximate ratio of 1:1. Consequently, a univariate Cox regression was first performed to filter out the DEGs that were not related to OS, and then 315 survival-related DEGs were identified. Based on the 315 survival-related DEGs, the relative regression coefficients were calculated by multivariate Cox regression with LASSO penalty. Using this method, we obtained seven potential prognostic genes, including APOLD1, C9orf66, G6PC, PPP1R1A, CNN1G, TIMP1, and TUBB2B (Fig. 2a; Table 1).

**Table 1:** Details of features selected by multivariate Cox proportional hazard regression model with LASSO penalty

Gene Name	Description	LASSO coefficient
APOLD1	Apolipoprotein L Domain Containing 1	−0.09978
C9orf66	Chromosome 9 Open Reading Frame 66	−0.01573
G6PC	Glucose-6-Phosphatase Catalytic Subunit	−0.06969
PPP1R1A	Protein Phosphatase 1 Regulatory Inhibitor Subunit 1A	0.02551
SCNN1G	Sodium Channel Epithelial 1 Subunit Gamma	0.01383
TIMP1	TIMP Metallopeptidase Inhibitor 1	0.13582
TUBB2B	Tubulin Beta 2B Class IIb	0.02215

A risk score (RS) was calculated for each patient in the TCGA training set by combining the relative expression of the DEGs in the prognostic model and the LASSO coefficients. Patients with an RS ≥0.323 (median cutoff) were classified as high risk and the remaining patients were classified as low risk, as shown in Fig. 2b. To investigate the relationship between RS and KIRC patients’ OS, a Kaplan–Meier analysis and log-rank test were performed using the training set. We found that high-risk patients had a worse prognosis than low-risk patients (Fig. 2c). The area under the curve (AUC) value was 0.738, as shown in the time-dependent receiver operating characteristic (ROC) curve assessing prognosis in Fig. 2d.

### Validation of the prognostic model using the TCGA and ICGC datasets

**Fig. 3:**
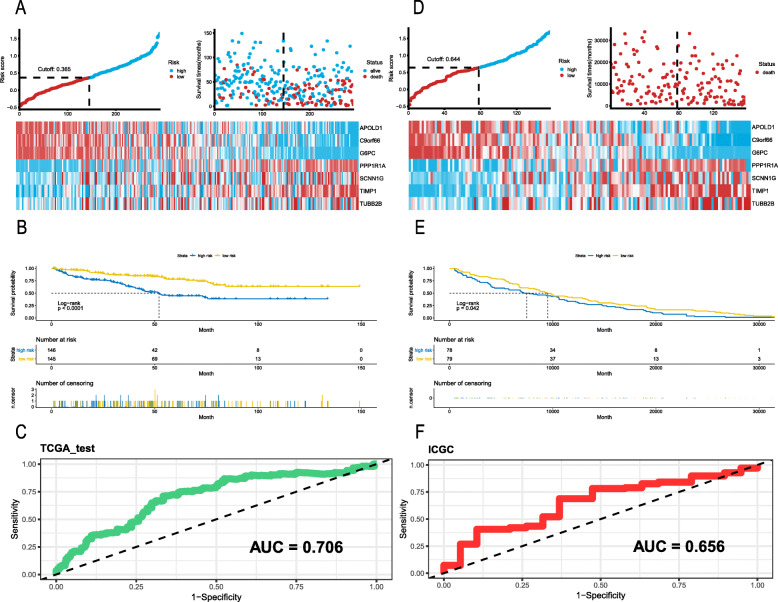
The distribution of RS, ROC curves and Kaplan-Meier survival in the testing and ICGC sets. **a**-**c**. Internal testing cohort. **d**-**f**. ICGC validation cohort

To further explore the relationship between RS and KIRC patients’ OS, a Kaplan–Meier analysis and log-rank test were performed on the TCGA and ICGC validation sets. In the TCGA validation set, we used the same prognostic model; patients with an RS ≥0.365 were classified as high risk and the remaining patients were classified as low risk by using the median of all risk scores (Fig. 3a). It is clear that the OS was significantly lower for patients with a higher RS than for patients with a lower RS (*P* < 0.0001; Fig. 3b). As most events occurred within 5 years, we used a time-dependent ROC curve to assess prognosis (Fig. 3c); the AUC value was 0.706. To verify that our prognostic model can be applied universally, we further applied the seven-gene signature to ICGC data. A total of 159 samples were obtained from the ICGC database, and after batch effect, 157 samples remained. Using the median cutoff of RS = 0.644 (Fig. 3d), the prognostic model successfully subdivided the patients into a high-risk group or a low-risk group, and the OS was significantly different. The five-year survival rate of patients in the high-risk group was low (Fig. 3e). The time-dependent ROC curve demonstrated an AUC of 0.656 (Fig. 3f), which showed better prediction performance. Moreover, we demonstrated the universal prognostic value of the seven-gene signature in the TCGA cohort despite the pathological stage, especially for stages I, III and IV (all *P* < 0.05, Table 2).

**Table 2:** Prognostic value of 7-gene signature in different pathological stages of KIRC in the TCGA cohort

	Number of samples	Number of Death (%)	Hazard Ratio	95% CI	***P*** Value
Stage I	214	35 (16)	5.16	2.07–12.87	0.0004
Stage II	42	7 (17)	1.15	0.11–11.54	0.908
Stage III	116	46 (40)	2.12	1.05–4.27	0.037
Stage IV	71	56 (79)	3.73	1.67–8.32	0.001

### Developing and validating a predictive nomogram based on the seven-gene prognostic model

**Fig. 4:**
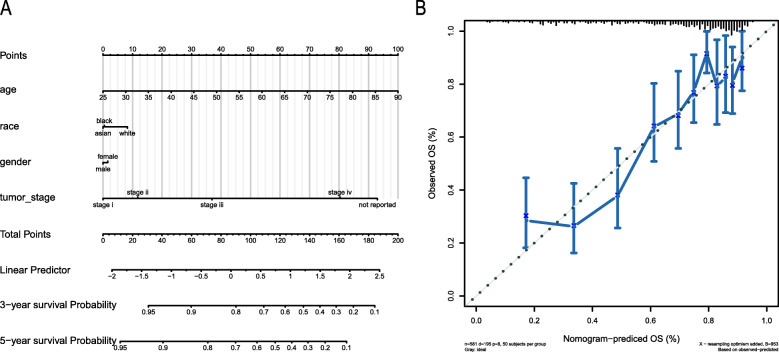
Nomogram for predicting 3- and 5-year OS. **a**. We added up the points identified on the points scale for each variable that can be projected onto the scales to indicate the probability of 3- and 5-year OS. **b**. Calibration plot showing the prediction of OS. The nomogram-predicted probability of OS is plotted on the x-axis; actual OS is plotted on the y-axis

To establish a survival prediction method for KIRC patients, a nomogram was used to predict the probability of three- and five-year OS in the TCGA cohort. The predictors in the nomogram included four independent prognostic factors (age, gender, tumour stage, and race (Fig. 4a) [14]. The calibration curve illustrated that the predictions and actual observations matched well, which indicated an accurate prediction via the nomogram (Fig. 4b) [15].

## Discussion

There is growing evidence that, despite the importance of individual molecules, tumorigenesis and prognosis are strictly controlled by interactions between a large number of cellular components including DNA, RNA, proteins, and small molecules [16]. However, the number of specific biomarkers with prognostic significance is still small [17], and the identification of prognostic factors is important for the optimal treatment of KIRC patients. Therefore, to reduce mortality and improve the prognosis of KIRC, molecular screening of KIRC biomarkers is urgently needed. In this study, we identified 333 DEGs by analysing GEO data. We then conducted a GO enrichment analysis, showing that the 333 DEGs are primarily involved in renal system development, kidney epithelium development, renal tubule development, and kidney development. After multivariate Cox regression with LASSO penalty, seven DEGs were identified, and two validation analyses were performed using independent datasets, showing good reproducibility.

The biological functions of the seven identified DEGs have been reported in previous studies. However, only a few of the DEGs have been investigated in KIRC. APOLD1 (Apolipoprotein L Domain Containing 1) is an endothelial cell early response protein that may play an important role in the regulation of endothelial signalling pathways and vascular function. C9orf66 (Chromosome 9 Open Reading Frame 66) is a protein-coding gene. G6PC (Glucose-6-Phosphatase Catalytic Subunit) is also a protein-coding gene. Any defects in this gene abrogate G6Pase function [18–20], which is associated with increased glycogen accumulation in gluconeogenic organs, especially in the kidneys, where it promotes progressive nephromegaly [21]. Poor metabolic control often results in long term complications such as renal dysfunction, pancreatitis, and hypertriglyceridemia, impairing kidney function and increasing the probability of KIRC [21]. PPP1R1A (Protein Phosphatase 1 Regulatory Inhibitor Subunit 1A) is a protein-coding gene [22]. TIMP1 (TIMP Metallopeptidase Inhibitor 1) is also a protein-coding gene [23]. The proteins encoded by this gene family are natural inhibitors of matrix metalloproteinases (MMPs). In addition to its inhibitory role against most of the known MMPs, TIMP1 promotes cell proliferation in a wide range of cell types and may also have an anti-apoptotic function. TUBB2B (Tubulin Beta 2B Class IIb) is a protein-coding gene. TUBB2B mutation leads to tubulin heterodimerization impairment, decreased ability to incorporate into the cytoskeleton, and alteration of microtubule dynamics, with an accelerated rate of depolymerization, which causes renal disease and an increase in the incidence of KIRC [24].

Compared to previous research, our study had some differences [25, 26]. First, our risk score (RS) strategy involved LASSO penalized regression which can analyse all independent variables as well as the most influential variables. When dealing with large datasets such as gene expression profiles, this method is much more accurate than the stepwise regression method of multivariate Cox regression models. Moreover, we used data from GSE8050, GSE12606, GSE14762, GSE36895, and GSE46699 KIRC expression profiling chips to identify DEGs and TCGA data for validation, and we then used ICGC data for external validation. We also acknowledge the limitations of this study. First, before clinical application, PCR-based sample validation should be conducted. Second, the functional phenotypes and mechanisms of the seven genes deserve further investigation. Third, a treatment effect that would influence patients’ prognosis was ignored when developing a prognostic model due to incomplete medical records.

## Conclusion

In summary, we developed a seven-gene signature that is associated with OS in KIRC patients. Our findings suggest that the seven-gene signature can serve as an independent biomarker for predicting survival prognosis, and we are poised for further investigation and eagerly anticipate the verification of our findings in a larger cohort of patients to assess whether the seven genes are likely to become new drug treatment targets.

## Methods

### KIRC sample sources

The following five KIRC expression profiling chip datasets, based on the GPL570 platform, were downloaded from the Gene Expression Omnibus (GEO) database: GSE8050, GSE12606, GSE14762, GSE36895, and GSE46699 with a total of 218 KIRC and normal kidney tissue samples. After removal of the samples with inadequate clinical information, 99 KIRC and 74 normal control samples were selected for this analysis. KIRC clinical and gene expression data (605 cases) were downloaded from the TCGA database, and a total of 591 cases ware obtained after removing the batch effect. This study strictly followed the published guidelines issued by TCGA. The TCGA data were randomly divided and used as a prognostic model training set and an internal testing set, and the ICGC data were used as an external validation set.

### Screening for differentially expressed genes (DEGs)

Differentially expressed genes (DEGs) were identified by R software and the screening criteria were absolute logFoldChange > 2 with adjusted *P* < 0.05. A total of 333 DEGs were identified between 99 KIRC and 74 normal control samples. These genes were then mapped to the TCGA and International Cancer Genome Consortium (ICGC) databases using the ID database. Excluding unmatched genes, 315 genes were available for analysis.

### Gene ontology (GO) enrichment analysis of DEGs

The biological significance of the DEGs was explored using a GO term enrichment analysis of biological processes, cellular components, and molecular functions. The search tool for recurring instances of neighbouring genes (STRING) [27] was used by inputting the gene name of each DEG and exporting the results [28].

### Screening for KIRC survival-related genes

We randomly divided the 591 TCGA samples with approximate ratio of 1:1 and 300 samples were set as the training set and 291 samples were set as the internal testing set. In the training group, multivariate Cox proportional hazard regression analysis was performed on 315 DEGs [29, 30], followed by LASSO penalty to further screen out a group of independent prognostic candidate genes with the strongest predictive power [31].

### Survival analysis

All statistical analyses were conducted by R3.6.2. Kaplan-Meier curves were generated for survival rates of patients, with difference detection based on log-rank testing. A Cox proportional hazard regression model was used to calculate the hazard ratios (HRs) and 95% confidence intervals (CIs) regarding OS [13]. Specifically, survival curves were established in the training set, internal testing set and ICGC set. The predictive performance of the nomogram was evaluated by a calibration curve [15]. For all statistical analyses, a two-tailed *P* value less than 0.05 was considered statistically significant.

## Data Availability

All analyzed data related to this paper are included in this paper.

## Abbreviations

KIRC: Kidney renal clear cell carcinoma
DEGs: Differentially expressed genes
GEO: Gene Expression Omnibus
TCGA: The Cancer Genome Atlas
ICGC: International Cancer Genome Consortium
OS: Overall survival
LASSO: Least absolute shrinkage and selection operator
ROC: Receiver-operator characteristic
AUC: Area under curve
STRING: Search Tool for Recurring Instances of Neighbouring Genes
RS: Risk score
RPPA: Reverse phase protein array
APOLD1: Apolipoprotein L Domain Containing 1
C9orf66: Chromosome 9 Open Reading Frame 66
G6PC: Glucose-6-Phosphatase Catalytic Subunit
PPP1R1A: Protein Phosphatase 1 Regulatory Inhibitor Subunit 1A
TIMP1: TIMP Metallopeptidase Inhibitor 1
MMPs: matrix metalloproteinases
TUBB2B: Tubulin Beta 2B Class IIb
